# Acupuncture De Qi in Stable Somatosensory Stroke Patients: Relations with Effective Brain Network for Motor Recovery

**DOI:** 10.1155/2013/197238

**Published:** 2013-06-02

**Authors:** Lijun Bai, Fangyuan Cui, Yihuai Zou, Lixing Lao

**Affiliations:** ^1^The Key Laboratory of Biomedical Information Engineering, Ministry of Education, Department of Biomedical Engineering, School of Life Science and Technology, Xi'an Jiaotong University, Xi'an 710049, China; ^2^Dongzhimen Hospital of Beijing University of Chinese Medicine, Beijing 100070, China; ^3^Center for Integrative Medicine, School of Medicine, University of Maryland, 520 W. Lombard Street, Baltimore, MD 21201, USA

## Abstract

Acupuncture has been widely used for treating stroke and De Qi may play an important role. In spite of its acceptance, the neural mechanism underlying acupuncture for motor recovery is still elusive. Particularly, by what extent De Qi sensations can reliably predict the therapeutical acupuncture effect on the mediating recovery from stroke is urgent to investigate. Nine stroke patients were assessed by De Qi, neurological examination, and scanned with acupuncture stimuli across two time points at an interval of two weeks. And we adopted multivariate Granger causality analysis to explore the interregional influences within motor executive brain network during post-acupuncture resting state. Our findings indicated that acupuncture at GB34 can enhance the recovery of stroke mainly by strengthening causal influences between the ipsilesional and contralesional motor cortex. Moreover, centrality of some motor-related regions correlated with clinical variables and thus served as a predictor of stroke recovery. Along the same line, the centrality of these motor-related regions has also high relations with the De Qi sensation. Our findings suggest that De Qi having relatively stable reliability may be essential and used as a predictor to the therapeutic effectiveness of acupuncture for stroke recovery.

## 1. Introduction 

Acupuncture is an ancient East Asian healing modality that has been in use for more than 2000 years. In the last decades, acupuncture has gained great popularity as an alternative and complementary therapeutic intervention in the Western medicine [1]. In this process, the boundaries between East Asian medicines and biomedicine/science are porous, negotiated to connect different medical traditions. De Qi, rooted in the central concept of the Traditional Chinese Medicine, was generally experienced by the patients and also by manipulating feeling of the acupuncturist when reaches the level of “Qi” in the body. Recently, researchers paid more attention to the patient's sensations rather than the acupuncturist's experience during acupuncture needling treatment [2–4]. However, consistent scientific investigation has found about neither histological nor physiological correlates for traditional Chinese concepts such as De Qi. 

According to the Traditional Chinese Medicine approach, stimulating specific acupuncture points corrects imbalances in the flow of Qi through channels known as meridians. During a typical acupuncture session, many practitioners perform needle manipulation in order to achieve the De Qi response. De Qi is believed to be essential to the therapeutic effectiveness of acupuncture and is often used as a signal to acupuncturists that the proper amount of needle stimulation is being performed [4–8]. It is also proposed that the patient's response (De Qi) served as a basis for “dose” of acupuncture needling, which calls for a better understanding of both the qualitative and quantitative characterization of the De Qi sensation [9, 10]. One recent report investigated the characteristics of the “De Qi” response in acupuncture at different acupoints (ST36, LI4, LV3) and its association with distinct nerve fibers, compared with the conventional somatosensory or noxious stimuli. They indicated that aching, soreness, and pressure were the most common sensations for different acupoints, followed by tingling, numbness, dull pain, heaviness, warmth, fullness, and coolness. And the sharp pain was regarded as inadvertent noxious stimulation. The most specific sensations of De Qi were aching, soreness, pressure and dull pain, in comparison with tactile stimulation control. Such complex composite of De Qi sensations indicated involvement of nerve fibers at all levels (myelinated and unmyelinated nerve fibers). Particularly, the deeper muscle layers with their rich supply of slow conducting fibers may play the key role in acupuncture. It is consistent with the findings that De Qi sensations are blocked after injection of procaine into the muscle beneath the acupoints. Following lumbar anesthesia, both De Qi sensations and electromyography were completely abolished [11]. This phenomenon inferred that acupuncture-induced sensations were mainly generated from muscle and the activity of polymodal-type receptors in deep tissues may play an important role [12]. Other researches find the relations of the functional effects of De Qi with the changes in skin resistance [12, 13], modification of different evoked potential parameters [14, 15], increases in the cortisone serum level [16], or remote functional modifications [17]. These findings partly provide a clue to demonstrate the De Qi with modern concepts in neurophysiology and bearing clinically relevance. 

Given that De Qi plays a pivot role in the therapeutic effect of acupuncture, some researches attempted to find out whether De Qi has any objective neurological correlate with the aid of the neuroimaging techniques. One study showed that acupuncture-induced De Qi sensations without sharp pain primarily elicited widespread signal decreases in several brain areas, including the frontal pole, ventral medial prefrontal cortex, cingulated cortex, hypothalamus, reticular formation, and cerebellar vermis, whereas sharp pain elicited signal increases in several areas including the frontal pole and anterior, middle, and posterior cingulate [18]. They further inferred that acupuncture feeling, without sharp pain is related to analgesia and antistress and deactivate the limbic-subcortical regions. By contrast, acupuncture feeling mixed with the sharp pain is associated with needling stimulation in deep tissue with skin piercing and biochemical reaction to tissue damage, and thus the central effects of pain prevailed, exhibiting an integrated response with predominance of activation over deactivation in the cerebrocerebellar and limbic systems. Another research indicated that individual differences in the De Qi scores can modulate the degree to which the right anterior insula was activated only following the verum acupuncture at ST36, compared with sham control [19]. The anterior insula has been widely accepted as a relay station integrating the centrally processed sensory information (visceral and autonomic) for its reciprocal connections with multiple brain regions [20]. This region, particularly the right anterior part, also plays a critical role in the interoceptive awareness of both stimulus-induced and stimulus-independent changes in the homeostatic state [21, 22], which enables us to regulate the organism's current state by initiating appropriate control signals toward the extrapersonal stimuli. This observation may suggest a key role of De Qi in characterizing the central expression of acupuncture stimulation at ST36, which is relevant to its clinical efficacy in gastrointestinal analgesia.

However, most of previous studies adopted healthy subjects and explored the neural mechanism of De Qi sensations mainly derived from the activation pattern of brain. Since acupuncture plays a homeostatic role it may have a greater effect on patients with a pathological imbalance compared to healthy controls [23, 24]. The hypothesis underlying neurobiological mechanism of De Qi needs further investigations in the altered and/or dysfunctional brain networks in patients. In addition, current neuroimaging studies focus on the spatial distribution of brain activity patterns induced by acupuncture. In fact, the well-identified physical effects of acupuncture needling and its purported clinical efficacy suggest that acupuncture acts in maintaining a homeostatic balance of the internal state within and across multiple brain systems [25]. Exploring the interactions between interregional effective connectivity and De Qi sensations modulated by acupuncture may provide a clue to understand the organizations of neural pathways underlying De Qi. More importantly, the reliability and reproducibility pattern of BOLD signal changes presents a significant challenge for both evaluating the effect and interpreting the neural mechanism of acupuncture. Although this discrepancy may partly be derived from different acupuncture modalities, needling dose, and postprocessing methods, individual physiology states contributes largely to such discrepancy. In the present study, we aim to address the above issues adopting the stable somatosensory stroke patients as the study cohort and using the individual-based Granger causality brain network analysis, in order to test the functional neurobiology of De Qi with two separate fMRI scannings as an interval of two weeks. In other words, we would like to determine whether, in acupuncture, the greater the individual sensorial needling experience, the greater interregional connectivity density, or whether such hypothesis can be verified by multiple measurements.

## 2. Materials and Methods

### 2.1. Subjects

A total of 9 patients (7 Males, mean age: 57.7 ± 9.92 years), recruited from Beijing Dongzhimen Hospital, were diagnosed with right hemispheric striatocapsular infarction and stable ischemic stroke by MRI with unilateral upper-limb disability. The criteria for patients recruitment are listed as follows: (1) stable recovery stroke patients: >2 weeks and <12 weeks after the onset of stroke (first episode of stroke); (2) sufficient cognition to follow simple commands, (minimental state examination score) MMSE >21. Patients were excluded if they met any of the below criteria: (1) bilateral infarcts, (2) recurrent stroke, (3) any previous history of alcohol or drug abuse, (4) history of epilepsy or other neurological disease and psychiatric disorder, (5) serious cognitive deficits, comprehensive aphasia and (6) other MRI contraindications (such as claustrophobia, etc.). The topographic distribution of the somatosensory deficit and the anatomic reconstruction of the brain lesions were shown in Table 1. Another 8 age-matched and sexually matched normal subjects (6 Males, mean ag: 51.6 ± 4.8 year) who were also recruited from Beijing Dongzhimen Hospital served as healthy controls. Each of them has normal neurological examination; no history of epilepsy or other neurological disease, psychiatric disorder and other MRI contraindications (such as claustrophobia, etc.). All of the patients and the normal subjects are with right-hand dominance.

### 2.2. Clinical Assessments

Each patient underwent a series of clinical evaluations. Clinical outcomes measurements included the National Institute of Health Stroke Scale (NIHSS), Ashworth Scale for clinical measure of muscle spasticity, Brunnstrom for sequential motor recovery, Rankin Scale For stroke disability, and Barthel Index of Activities of Daily Living and Motricity Index. The anatomic reconstructions of the brain lesions are listed in Table 1. One patient had only taken part in the first scanning and the second clinical assessments and scanning. 

### 2.3. fMRI Motor Task

**Figure 1:**
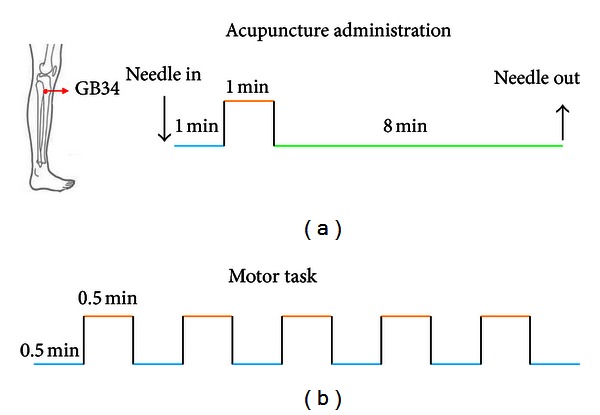
Acupuncture stimuli and motor task experimental paradigm.

During fMRI scanning, a simple finger movement was firstly served as stimulation for patients. A simple block design was performed in which 30-second baseline and 30-second stimulation alternated and lasted for 5 minutes and 30 seconds, with 10 seconds rest in the beginning. And the healthy subjects had the same MRI procedure as the patients (Figure 1).

### 2.4. Acupuncture Stimulation

A multiblock paradigm is generally used in acupuncture fMRI studies, which implicitly presumes the temporal intensity profiles of the certain event conforming to the “on-off” specifications. Since the acupuncture action is slow to develop and resolve [25], the temporal aspects of the BOLD response to acupuncture may violate the assumptions of the block-designed estimates. In addition, using several stimulation blocks in a short period of time, investigators may not be able to dissociate the long-lasting effects from other confounding changes, such as the effect of needle manipulation during the experiment [26]. In the current study, we adopted a new experimental paradigm, namely, the nonrepeated event-related fMRI (NRER-fMRI) design, to investigate such prolonged effects after acupuncture administration.

Acupuncture stimulation employed the NRER-fMRI design paradigm scanning, incorporating 1 min. needle manipulation, preceded by 1 min. rest epoch, and followed by 10 min. rest (without acupuncture manipulation) scanning (Figure 1). Acupuncture was performed at acupoint GB34 on the left leg (located in the lateral aspect of the posterior knee). According to the TCM, the first choice acupoint for stroke is located at the scalp. Considering both limitation of fMRI scanning and classic use, we selected Yangming channel for Wei syndrome. GB34 is one of the most frequently used acupoints and proved to have various efficacies in the treatments of hemiplegia and rehabilitation for motor functional deficit/impairment after stroke. Acupuncture stimulation was delivered using a sterile disposable 38 gauge stainless steel acupuncture needle, 0.2 mm in diameter and 40 mm in length. The needle was inserted vertically to a depth of 2-3 cm, and the administration was delivered by a balanced “tonifying and reducing” technique. The stimulation consisted of rotating the needle clockwise and counterclockwise for 1 min. at a rate of 150 times per min. The procedure was performed by the same experienced and licensed acupuncturist on all participants. Every subject endured twice acupuncture stimulation scanning at an interval of two weeks, in order to test the reliability of the hypothesis.

At the end of each fMRI scanning, the subjects completed a questionnaire that used a 10-point visual analogue scale (VAS) to rate their experience (or “De Qi”) of aching, pressure, soreness, heaviness, fullness, warmth, coolness, numbness, tingling, dull, or sharp pain they felt during the scan. The VAS was scaled at 0 = no sensation, 1–3 = mild, 4–6 = moderate, 7-8 = strong, 9 = severe, and 10 = unbearable sensation [16]. The questionnaire also had one blank row for subjects to add their own words if the above descriptors did not embody the sensations they experienced during the stimulation. Because sharp pain was considered an inadvertent noxious stimulation, we excluded the subjects from further analysis if they experienced the sharp pain (greater than the mean by more than two standard deviations). Among the sixteen participants, none experienced the sharp pain.

### 2.5. Imaging Data Acquisition and Analysis

The images were acquired on a 3T Siemens MRI Scanner. A custom-built head holder was used to prevent head movements. Thirty-two axial slices (FOV = 225 mm × 225 mm, matrix = 64 × 64, thickness = 3.5 mm) parallel to the AC-PC plane and covering the whole brain were obtained using a T2*-weighted single-shot, gradient-recalled echo planar imaging (EPI) sequence (*T*_*R*_ = 2000 ms, *T*_*E*_ = 30 ms, flip angle = 90°). Prior to the functional run, high-resolution structural information on each subject was also acquired using 3D MRI sequences with a voxel size of 1 mm^3^ for anatomical localization (*T*_*R*_ = 1.9 s, *T*_*E*_ = 2.52 ms, matrix = 256 × 256, FOV = 250 mm × 250 mm, flip angle = 9°, and slice thickness = 1 mm). 

All preprocessing steps were carried out using statistical parametric mapping (SPM5, http://www.fil.ion.ucl.ac.uk/spm/). The images were first slice-timed and then realigned to correct for head motions (none of the subjects had head movements exceeding 1 mm on any axis and head rotation greater than one degree). The image data was further processed with spatial normalization based on the MNI space and resampled at 2 mm × 2 mm × 2 mm. Finally, the functional images were spatially smoothed with a 6 mm full-width-at-half maximum (FWHM) Gaussian kernel. The statistics were color-coded and mapped in Talairach space. 

For motor task, statistical analysis was performed in two steps. First, a single subject fixed effects model was used. The difference between the motor condition and the resting was estimated at each voxel by using the general linear model (GLM) and the parameter estimates for the covariate resulting from the least mean square fit of the model to the data were calculated. In the second-level analysis, the obtained individual *t*-maps were used in “random effect” group analysis framework by one-sample *t*-test for different groups. The statistical threshold was set at *P* < 0.05 (corrected for multiple comparisons). 

### 2.6. ROI Definition

According to previous imaging studies on poststroke brain organization, motor execution areas rather than motor preparation areas play a key role [27]. We selected the regions of interest associated with the motor execution network from motor task by healthy controls (*P* < 0.01, FDR corrected). The regions of interest included regions, such as bilateral primary motor cortex, bilateral dorsolateral and ventrolateral premotor cortex, bilateral superior parietal lobule, bilateral basal ganglia, bilateral thalamus, anterior inferior cerebellum, left postcentral gyrus, and supplement motor area. In order to refine the accuracy of the ROIs, several procedures were conducted: the effect of intersubject anatomical variability was examined by defining ROIs in individual anatomical space, group-probabilistic anatomical map, as well as using the standard Talairach-Daemon-based atlas [28]. In order to obtain the group-probabilistic anatomical map, the individually drawn ROIs were registered to standard MNI space [28] and summed across all subjects

### 2.7. Effective Connectivity Networks Generated from mGCA

The entire time series of BOLD signal intensities from these selected ROIs during the PARS and PSRS, averaged across voxels within each ROI, were normalized across subjects (separately for different conditions) to form a single vector per ROI. The mGCA used the directed transfer function (DTF) [29], computed from a multivariate autoregressive model of the time series in the selected ROIs. In this study, we also adopted the weighted DTF with partial coherence in order to emphasize direct connections and inhibit mediated influences [30, 31]. To assess the significance of path weights, a null distribution was obtained by generating 2500 sets of surrogate data and calculating the DTF from these 2500 datasets [29, 30, 32]. The DTF value was compared with the null distribution for a one-tailed test of significance with a *P* value of 0.01 (corrected for multiple comparisons).

In order to better extract information on the temporal relations among the regions obtained from mGCA, a node interaction analysis was performed. In-degree of a node in a Granger causal connectivity network means the number of causal in-flow connections to the node from any of the other nodes. Out-degree of a node means the number of causal out-flow connections from the node to any of the other nodes in the network. We calculated “In + Out degree” for every node within the DMN, respectively. The region was identified as the hub in the network if its sum of “In degree” and “Out degree” was at least one standard deviation (SD) greater than the average “In + Out degree” for all regions (i.e., sum > mean + SD).

### 2.8. Subjective Acupuncture Sensations Analysis

Because sharp pain was considered an inadvertent noxious stimulation, we excluded the subjects from the further analysis if they experienced the sharp pain (greater than the mean by more than two standard deviations). Among the thirty-two participants, none experienced the sharp pain. In order to quantify the total intensity of De Qi experienced by each individual, we employed the MGH Acupuncture Sensation Scale (MASS) index, defined as a weighted average of all sensations using an exponential smoothing [33]. This index is convenient to devise a single value to quantitatively summarize the full multivariate breadth and depth of acupuncture sensations.

## 3. Results

### 3.1. Psychological Analysis from De Qi Sensations

The prevalence of these sensations was expressed as the percentage of individuals in the group that reported the given sensations. A statistical analysis found significant difference between the stroke and healthy control with regard to the prevalence of these sensations (*P* < 0.05). For stroke patients, fullness and numbness were most common sensations, while fullness and aching were most common sensations for healthy controls. The intensity of sensations was expressed as the average score ± SE. The levels of sensations were kept low (mild to moderate), but statistically significant differences occurred in the average sensation intensity between the stroke patients (3.7 ± 1.2) and healthy controls (2.1 ± 1.4). In addition, the variability of De Qi between the first and second scanning at an interval of two weeks kept relatively stable for both intensity and prevalence (*P* > 0.09).

### 3.2. MGCA Mapping for Stroke Patients and Healthy Controls

**Figure 2:**
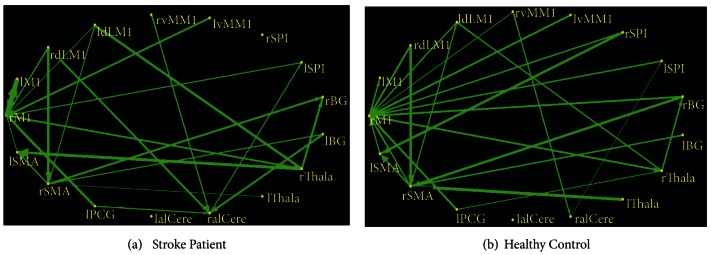
Multivariate Granger causality connectivities among selected ROIs (*P* < 0.01, corrected for multiple comparisons) for both stroke patients and healthy controls during the postelectroacupuncture resting state. Relative strengths of path weights (in arbitrary units) were indicated by the width of arrows. M1: premotor cortex; dlM1: dorsolateral premotor cortex; vMM1: ventrolateral premotor cortex; SPl: superior parietal lobule; BG: basal ganglia; Thala: thalamus; aICere: anterior inferior cerebellum; PCG: postcentral gyrus; SMA: supplement motor area; l: left; r: right.

A causal connectivity graph was constructed using the thickness of connecting arrows to indicate the strengths of the causal influences (shown in Figure 2). For stroke patients, the right premotor cortex, the right supplement motor area and the right anterior inferior cerebellum served as the hub, and were central targets during the post-acupuncture resting period. For normal controls, the central targets remain the same, while the brain network has more dense effective connectivities. Among causal influences of each node, the right premotor cortex projected the strongest inflow into the left premotor cortex, and also became even stronger than that of the normal controls. While, paths originating from the right motor cortex to bilateral basal ganglia and right superior parietal lobule became weak below the statistical significance level for stroke patients. 

At an interval of two weeks, the interregional causal influence pattern kept relatively stable during the post-acupuncture resting epoch; however, the “in + out” degree in the right premotor cortex, right supplement motor area and the left postcentral cortex became much higher and more saliently for stroke patients. On the other hand, a trend towards a significant decrease was detected in the “in + out” degree in the right anterior inferior cerebellum and the right thalamus. Specifically, the causal interactions between the right premotor cortex and left premotor cortex attenuated in the stroke patients during the post-acupuncture resting period. However, the interregional causal influences among the ipsilateral motor cortices (the premotor cortex, the supplement motor area as well as the postcentral cortex) became even stronger. Path originating from the right ventrolateral premotor cortex projecting to the right thalamus as well as path from the anterior inferior cerebellum to the superior parietal lobule became decreased strengths. 

### 3.3. Relationship between Network Parameters and the Clinical Measures

**Figure 3:**
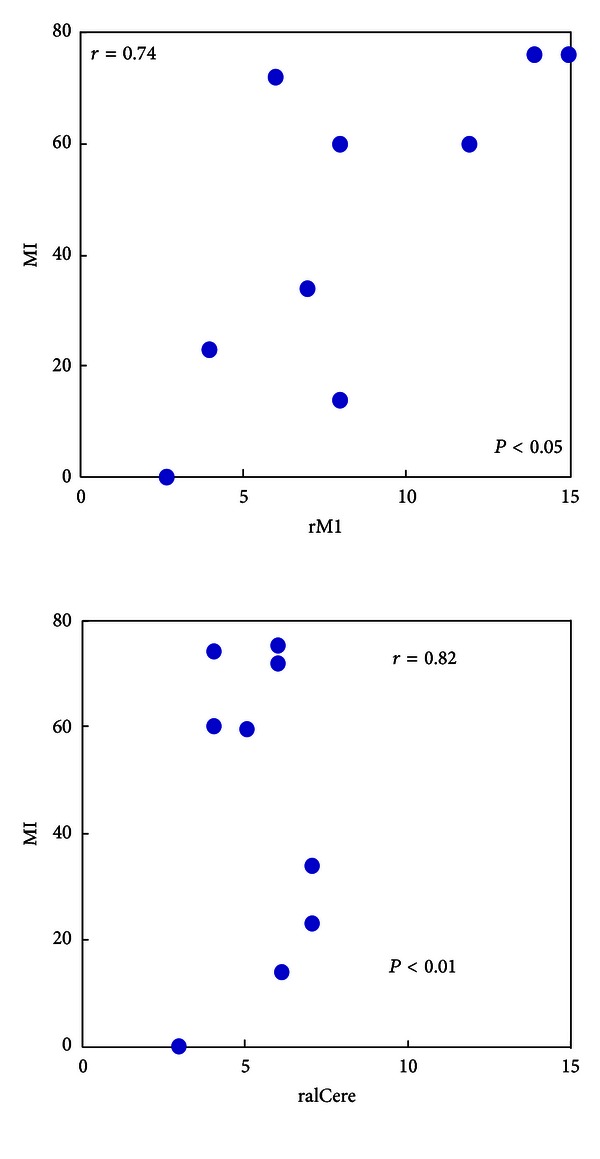
Cross subject correlation between the “in + out” degree of brain areas and Motor Index (MI) for stroke patients. For stroke patients, the MI and “in + out” degree of the right premotor cortex (rM1) presented the significantly positive relation (*r* = 0.74, *P* < 0.05, *n* = 9), while the significant negative correlation was mainly exhibited in the “in + out” degree of the anterior inferior cerebellum (*r* = −0.82, *P* < 0.01, *n* = 9).

**Figure 4:**
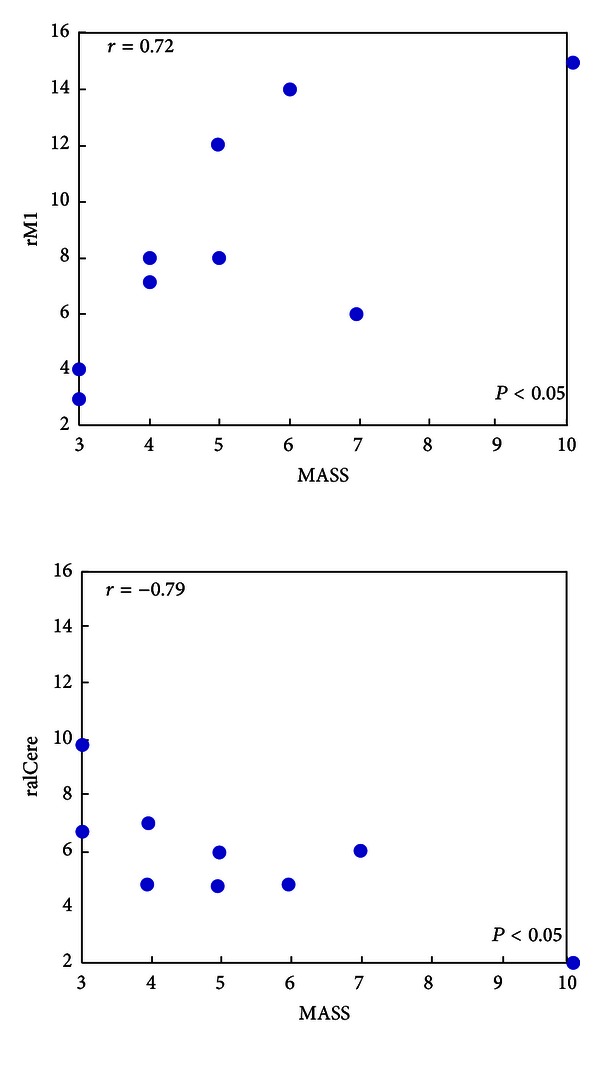
Cross subject correlation between the “in + out” degree of brain areas and MGH Acupuncture Sensation Scale (MASS) for stroke patients. For stroke patients, the MASS and “in + out” degree of the right premotor cortex (rM1) presented the significantly positive relation (*r* = 0.72, *P* < 0.05, *n* = 9), while the significant negative correlation was mainly exhibited in the “in + out” degree of the anterior inferior cerebellum (*r* = −0.79, *P* < 0.05, *n* = 9).

To test relationships between the nature of regional centrality (“in + out degree of certain node”) and neurological scales, we calculated the cross subject correlation between the “in + out” degree of certain node and the Motricity Index, Barthel Index, the National Institute of Health Stroke Scale, Ashworth Scale, Rankin, and Brunnstrom Scale (Figures 3 and 4). Our results presented that the centrality of the right premotor cortex indicated a significantly positive relation with the Motricity Index (*r* = 0.74, *P* < 0.05). By contrast, the centrality of the anterior inferior cerebellum showed the negative relation with the Motricity Index (*r* = −0.82, *P* < 0.01). Other correlations were not significant (*P* > 0.08). 

### 3.4. Relationship between Network Parameters and MASS Index

To test relationships between the nature of regional centrality (“in + out degree of certain node”) and De Qi sensations, we calculated the cross-subject correlation between the “in + out” degree of certain node and MASS (total intensity of De Qi sensations experience by each individual). Our results presented that the centrality of the right premotor cortex indicated a significantly positive relation with the MASS (*r* = 0.72, *P* < 0.05). By contrast, the centrality of the anterior inferior cerebellum showed the negative relation with the MASS (*r* = −0.79, *P* < 0.05). Other correlations were not significant (*P* > 0.1). 

## 4. Discussion

The present study examined the relations of the De Qi sensations with the causal interactions within the motor executive brain network induced by acupuncture at GB34 for stable somatosensory stroke patients, compared with the healthy controls. 

The aim of this study was to address (i) whether De Qi sensation induced by acupuncture at GB34 is associated with heterogeneous motor-executive pathway for stroke patients and healthy controls; (ii) by what way acupuncture-induced effect may enhance recovery for stroke patients by activating motor-related brain networks? (iii) by what extent De Qi sensations can reliably predict the therapeutical acupuncture effect on the mediating recovery from stroke? Our findings demonstrated that acupuncture at GB34 can enhance the recovery of stroke mainly by strengthening the causal influences between the ipsilesional motor cortex and contralesional motor cortex. Moreover, the centrality of some motor-related regions correlated with clinical variables and thus served as a predictor of stroke recovery. Along the same line, the centrality of these motor-related regions has also high relations with the De Qi sensation. Collectively, our findings suggesting that De Qi has relatively stable reliability may be essential and used as a predictor to the therapeutic effectiveness of acupuncture for stroke recovery.

Most neuroimaging studies have focused on the spatial distribution of neural responses and more or less harness the exploration of brain networks underlying acupuncture. In addition to functional connectivity, effective connectivity between different regions is both important and essential in detailing working mechanisms of the brain's functional architecture underlying acupuncture effect. The resultant modeled primarily concern with the directions of neural interactions and how one neural system exerts influence over another. Granger causality has been highlighted in recent years, and is proved suitable for the study of directionality in neuronal interactions through assessment on neurophysiologic data in both the frequency and time domains [34–36]. It is assumed that the autoregressive prediction of the first time series at present time could be improved by including the past information of the second time series if the second time series has a causal influence on the first. The MGCA mapping showed that delayed effects of acupuncture exert distinct modulatory causal influence on motor-executive brain networks.

Acupuncture at GB34 can induce more enhanced bidirectional causal influence between the ipsilesional and contralestional premotor cortex for stroke patients, compared with the healthy controls. Accumulating evidence suggested that the outgrowth of enhanced connections may compensate for impaired neural pathways connecting important nodes after the motor pathway stroke. Cortical regions in the intact hemisphere are thought to be important in supporting motor function of the paretic hand after stroke. Contralesional premotor cortex is more active during the movement of the affected hand after stroke compared with that in healthy controls [37, 38], particularly for more impaired patients [39] with greater corticospinal tract disruption [40]. One research from animals has also indicated increased connections from the premotor cortex to the somatosensory cortex in a monkey with an ischaemic lesion to motor cortex [41]. This can be interpreted to indicate that stimulation of acupoints, used therapeutically, may enhance recovery from stroke selectively through improving the effective connectivities between these areas, which are generally thought to be involved in mediating recovery from stroke via functional plasticity. On the other hand, we also found that at an interval of two weeks, acupuncture at GB34, can induce decreased bidirectional causal influence between the ipsilesional and contralestional premotor cortex for stroke patients, compared with the first acupuncture administration. It is partly consistent with previous studies that neural activity in ipsilesional and contralesional cortical areas was pathologically increased when stroke patients moved their paretic hand, and that overactivity usually decreases over time, concomitant to clinical recovery [42, 43]. Another research also indicated that increased activation in the intact hemisphere is prominent in patients with poor motor recovery [40], and its also simply reflect the removal of transcallosal inhibition from the damaged hemisphere [44]. Our findings demonstrated that acupuncture at GB34 comply with the reorganization of brain networks and enhance the recovery of stroke patients. 

Notably, we found that the De Qi sensations induced by acupuncture at GB34 have relative relations with the regional centrality of motor-executive brain networks which can be used as the predictor for the motor recovery of stroke patients. Particularly, as patients demonstrated recovery from stroke, gradual increases in regional centrality were presented in the ipsilesional primary motor areas while the opposite change was seen in ipsilesional anterior inferior cerebellum. One research focusing on the acupuncture effect for facial muscle recovery has demonstrated that stronger intensity of De Qi was associated with better therapeutic effects including reduced disability and better quality of life [45]. Abundant evidence indicated that the increasing importance of ipsilesional primary motor areas as a key node is attributed to the recovery of stroke patients [46, 47]. Specifically, there is a general trend for the centralization of brain activity towards the primary motor area of lesion hemisphere as the time delayed. On the other hand, the centrality of the right anterior inferior cerebellum attenuated by acupuncture at GB34 after an interval of two weeks, which is also significantly correlated with the degree of individual motor ability. It is already proved that the large involvement of the ipsilesional cerebellum in the acute phrase of stroke is partly due to the overuse of the unaffected limbs and may result in the more focusing on this region. As the time passed and improving the motor ability of the affected limbs, the node centrality of the ipsilesional anterior inferior cerebellum is attenuated. This hypothesis can also be verified by the negative correlations between the regional centrality of this area and behavior recovery index. As a whole, acupuncture can provide beneficial effect on the recovery of stroke patients from subcortical lesions by coordinating the reorganization of motor-related brain network and enhancing the converging of certain regions, which is also predicted by the De Qi sensations experienced by patients enduring acupuncture treatments.

## Figures and Tables

**Table 1:** Clinical and demographic data.

	Patient number								
	1	2	3	4	5	6	7	8	9
Age (years)	56	64	57	68	57	37	58	71	52
Gender	F	M	M	M	F	M	M	M	M
Localization of infarct	BG	IC	IC	CR	IC	IC	IC	IC	BG
Motricity Index	0	60	14	72	23	60	34	76	76
	11	64	14	72	23	60	34	76	—
Rankin Scale	4	1	2	2	4	2	3	2	2
	4	1	2	1	4	2	3	1	—
Barthel Index	35	95	60	90	60	85	65	90	85
	40	95	65	85	60	85	75	90	—
NIHSS	14	3	9	5	8	7	7	3	5
	8	1	9	2	8	7	7	2	—
MMSE	22	30	27	29	22	30	30	24	30
	23	30	30	28	24	30	30	27	—
Brunnstrom	I	IV	II	II	I	V	II	V	II
	I	IV	II	III	I	V	II	V	—
Asworth	0	1	1	0	0	2	2	0	0
	0	1	0	1	0	2	2	0	—

BG: basal ganglia; IC: internal capsule; CR: corona radiate; NIHSS: National Institute of Health Stroke Scale; MMSE: minimental state Examination.
